# LONGTERM OFF-LABEL USE OF SSRI IN TREATMENT OF EARLY EJACULATION: TWO CASES REPORT

**DOI:** 10.1192/j.eurpsy.2023.2330

**Published:** 2023-07-19

**Authors:** E. Becirovic, A. Becirovic

**Affiliations:** 1 Klinika za psihijatriju; 2 UKC Tuzla, Tuzla, Bosnia and Herzegovina

## Abstract

**Introduction:**

Early ejaculation is besides erectile dysfunction the most common exual dysfunction amog males. It can create suffering and inflence relationships. Selective serotonin reuptake inhibitors has known side effect and sometimes are used as treatment for early ejaculation.

**Objectives:**

To present successful off-label treatment of early ejaculation.

**Methods:**

Case report. Two cases.

**Results:**

Two male patientes with early ejaculation problems that influenced their romatic and sexual life. Older one of them has devorced because of that. Younger has never had romantic or sexual relationship for the same reason. Prescribing SSRI make their love life more productiv. Older one married for the second time and has functional and stabile relationship. Younger one established several romantic and sexualn relatinship and currently is in a stabile romantic relationship.

**Conclusions:**

Longterm use of low doses SSRI can be very beneficial in treatment of early ejaculation.

**Disclosure of Interest:**

None Declared

